# Multi-Gene Prognostic Signatures and Prediction of Pathological Complete Response to Neoadjuvant Chemotherapy in ER-Positive, HER2-Negative Breast Cancer Patients

**DOI:** 10.3390/cancers12051133

**Published:** 2020-05-01

**Authors:** Claudia Mazo, Stephen Barron, Catherine Mooney, William M. Gallagher

**Affiliations:** 1 UCD School of Computer Science, University College Dublin, Dublin 4, Ireland; claudia.mazovargas@ucd.ie (C.M.); catherine.mooney@ucd.ie (C.M.); 2 CeADAR: Centre for Applied Data Analytics Research, University College Dublin, Dublin 4, Ireland; 3 OncoMark Limited, NovaUCD, Dublin 4, Ireland; stephen.barron@oncomark.com; 4 UCD School of Biomolecular and Biomedical Science, UCD Conway Institute, University College Dublin, Dublin 4, Ireland

**Keywords:** breast cancer, multi-gene prognostic signature, neoadjuvant chemotherapy, breast cancer treatment, pathological complete response

## Abstract

Determining which patients with early-stage breast cancer should receive chemotherapy is an important clinical issue. Chemotherapy has several adverse side effects, impacting on quality of life, along with significant economic consequences. There are a number of multi-gene prognostic signatures for breast cancer recurrence but there is less evidence that these prognostic signatures are predictive of therapy benefit. Biomarkers that can predict patient response to chemotherapy can help avoid ineffective over-treatment. The aim of this work was to assess if the OncoMasTR prognostic signature can predict pathological complete response (pCR) to neoadjuvant chemotherapy, and to compare its predictive value with other prognostic signatures: EndoPredict, Oncotype DX and Tumor Infiltrating Leukocytes. Gene expression datasets from ER-positive, HER2-negative breast cancer patients that had pre-treatment biopsies, received neoadjuvant chemotherapy and an assessment of pCR were obtained from the Gene Expression Omnibus repository. A total of 813 patients with 66 pCR events were included in the analysis. OncoMasTR, EndoPredict, Oncotype DX and Tumor Infiltrating Leukocytes numeric risk scores were approximated by applying the gene coefficients to the corresponding mean probe expression values. OncoMasTR, EndoPredict and Oncotype DX prognostic scores were moderately well correlated according to the Pearson’s correlation coefficient. Association with pCR was estimated using logistic regression. The odds ratio for a 1 standard deviation increase in risk score, adjusted for cohort, were similar in magnitude for all four signatures. Additionally, the four signatures were significant predictors of pCR. OncoMasTR added significant predictive value to Tumor Infiltrating Leukocytes signatures as determined by bivariable and trivariable analysis. In this *in silico* analysis, OncoMasTR, EndoPredict, Oncotype DX, and Tumor Infiltrating Leukocytes were significantly predictive of pCR to neoadjuvant chemotherapy in ER-positive and HER2-negative breast cancer patients.

## 1. Introduction

Breast cancer is the most frequently diagnosed cancer in women, with more than 2.1 million new diagnoses worldwide every year, and the second leading cause of cancer death. The American Cancer Society and the International Agency for Research on Cancer reported that mortality rates are on the decline in certain regions of the world as a result of earlier diagnosis and improved therapies [1,2]. Accurate tools to help with optimal treatment decisions for individual patients to improve their prognosis, survival and quality of life are needed, whilst also reducing associated healthcare costs [3]. Breast cancer patients who receive chemotherapy can experience several side effects and symptoms that have a negative effect on their quality of life during and after the completion of treatment [4]. Biomarkers that can predict patient response to chemotherapy can help identify which patients are likely to benefit from chemotherapy, thereby potentially reducing the adverse effects of over-treatment.

Multi-gene prognostic signatures may be used to estimate risk of recurrence following surgery and endocrine treatment to make decisions about the suitability of chemotherapy. A patient who is predicted to be at low risk of breast cancer recurrence can safely forego aggressive treatment plans such as chemotherapy. OncoMasTR (OM) is a new 6-gene assay (3 prognostic genes plus 3 reference genes) discovered using a novel transcriptional network analysis approach that identified genes—Master Transcriptional Regulators (MTRs)—that putatively regulate previously known prognostic signatures [5,6]. OM has been analytically validated [7] in terms of assay robustness and clinically validated [8,9] in terms of accurate risk stratification, providing significant additional prognostic value to clinical information. The Oncotype DX Recurrence Score (RS) is a 21-gene prognostic assay that is widely used to predict risk of recurrence [10]. The EndoPredict assay (EP) is a 12-gene prognostic assay which incorporates tumor size and nodal status [11]. The Tumor Infiltrating Leukocytes (TILs) signature [12] determines risk of recurrence using expression correlates of 60 genes representing 15 immune cell sub-populations. Elevated TILs levels have been associated with better survival in patients with either ER-positive, HER2-positive disease or Triple-Negative Breast Cancer (TNBC) [13,14,15,16,17]. Furthermore, high TILs levels are associated with increased sensitivity to chemotherapy, reflected by higher pCR rates to neoadjuvant chemotherapy [18,19]. Indeed, a critical question remains as to whether one can predict a particular benefit from chemotherapy, as opposed to solely prognostic information, in breast cancer patients using multi-gene signatures.

The focus of this work is on ER-positive, HER2-negative breast cancer patients as the pCR to neoadjuvant chemotherapy is normally quite low in these patients compared to other breast cancer subtypes. We present an approach to expand on previous *in silico* analyses of other multi-gene prognostic signatures [20]. We assessed if OM could predict pCR of early-stage breast cancer patients to neoadjuvant chemotherapy and subsequently compared its predictive performance with EP, RS and TILs. Additionally, we assessed if TILs added significant predictive value to RS, EP and OM using univariable, bivariable and trivariable logistic regression analysis.

## 2. Method

First, a systematic search was carried out to select the datasets for this study. Following this, the relationship between OM, RS, EP, and TILs standardised risk scores was assessed by the Pearson’s correlation coefficient [21,22]. Finally, logistic regression [23] was used to estimate the associations between the signatures and pCR.

### 2.1. Systematic Search

The following sources were used to identify relevant breast cancer datasets: (i) dataset engines such as the Gene Expression Omnibus (GEO) repository [24], ArrayExpress [25], GDC data portal [26] and EGAS [27]; (ii) related papers and similar studies, as well as peer reviewed papers that performed a systematic search for gene expression datasets in breast cancer.

**Figure 1:**
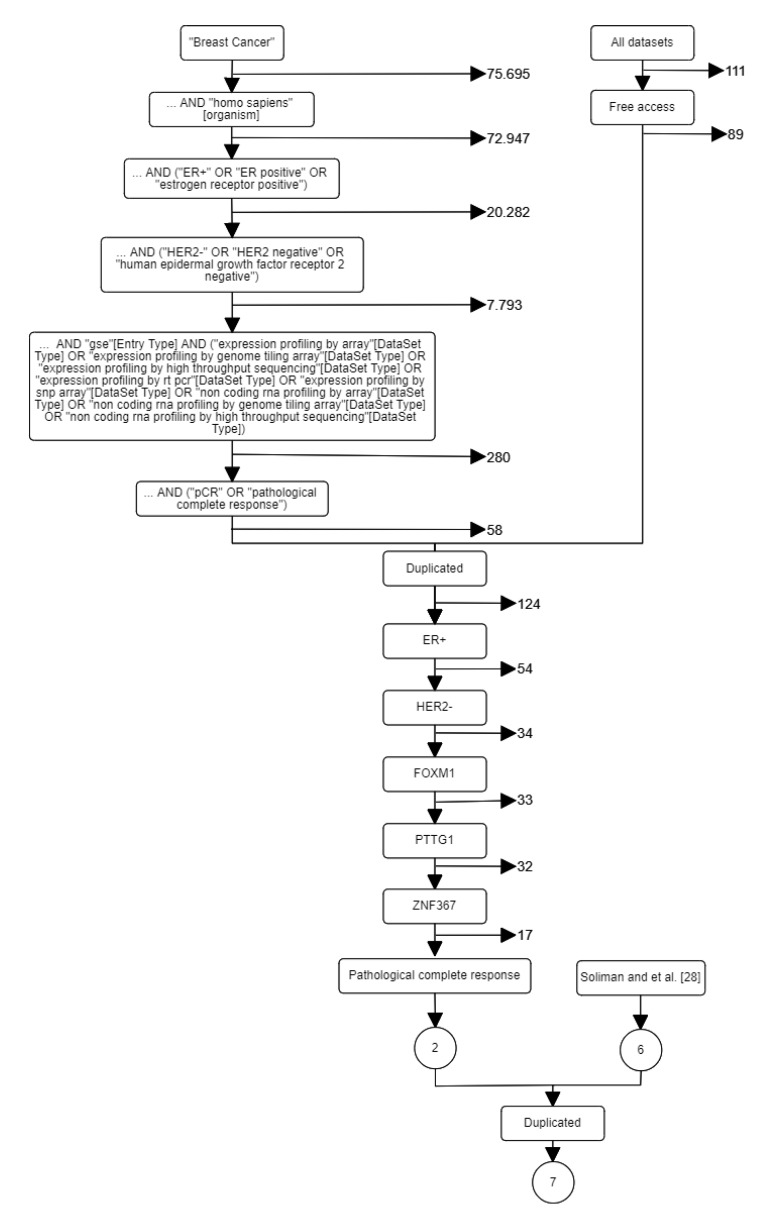
A systematic search for breast cancer datasets. Left path corresponds to GEO search. Right path corresponds to related papers search.

After the resources were specified, the following key words were selected: breast cancer, early, *Homo sapiens*, ER+, and HER2-. Particularly in GEO, there are many keywords and hierarchical orders that may be used in a systematic search resulting in different results. Furthermore, including the synonyms of the keywords or GEO’s query fields may alter the results obtained. Additional constraints were applied according to our project’s need: (i) including FOXM1, PTTG1, and ZNF367 genes; (ii) endpoint or outcome (e.g., pCR, distant recurrence, death, response to therapy); (iii) number of endpoint events; (iv) lymph node status; (v) therapy/treatment (e.g., none, endocrine, chemotherapy) (vi) platform; and (vii) public access. Figure 1 shows the flow diagram according to the project’s objectives after different search criteria. Finally, joining the results from the systematic search and the reference-work presented in [28], seven datasets (GSE16716, GSE20271, GSE25066, GSE32646, GSE34138, GSE41998, and GSE22226) were identified (see Table 1).

### 2.2. Dataset

Seven GEO dataset were used—GSE16716, GSE20271, GSE25066, GSE32646, GSE34138, GSE41998, and GSE22226. OM, EP and RS numeric risk scores were approximated by applying the signatures’ gene coefficients to the mean of the corresponding probe expression values. TILs scores is computed as the simple average log expression of their marker genes. Risk scores were standardised within each dataset to have a mean of 0 and a standard deviation of 1. This allowed us to compare different risk scores from different datasets on a similar scale. A total of 813 patients with 66 pCR events (8.1%) were analysed. All the datasets except GSE32646 were missing at least one gene from at least one signature (see Table 1).

### 2.3. Predicting Probability of pCR Using Logistic Regression Analysis

Logistic regression is a statistical model that uses a logistic function to model a binary dependent variable. Logistic regression is used for different scenarios; in this case to estimate the probability that an event will occur, the outcome, using information thought to influence or be related to such events called predictors [23]. The outcome is a binary variable (1/0, Yes/No, True/False). The odds are defined as the probability that the event will occur divided by the probability that the event will not occur. Odds > 1 shows an association or correlation between risk scores and pCR, in our case. The 95% Confidence Interval (CI) is used to estimate the precision of the odds. Additionally, a *p*-value is used as probability measure. The smaller the *p*-value (p) is the more significant the result is considered to be. 0.05 is chosen as an arbitrary threshold where *p*>0.05 is considered not to be significant. Univariable analysis was used to examine the relationship between pCR and each risk score while multivariable analysis was used to examine the relationship between pCR and each risk score, adjusted for cohort/GSE dataset or signature.

## 3. Results

### 3.1. Correlation Analysis

A Pearson’s correlation coefficient was used to assess the linear relationship between the standardised scores from the four signatures. We evaluated the correlation between six pairs of the four signatures: OM vs. RS, OM vs. EP, RS vs. EP, TILs vs. OM, TILs vs. EP, and TILs vs. RS for the seven datasets combined (Table 2).

**Figure 2:**
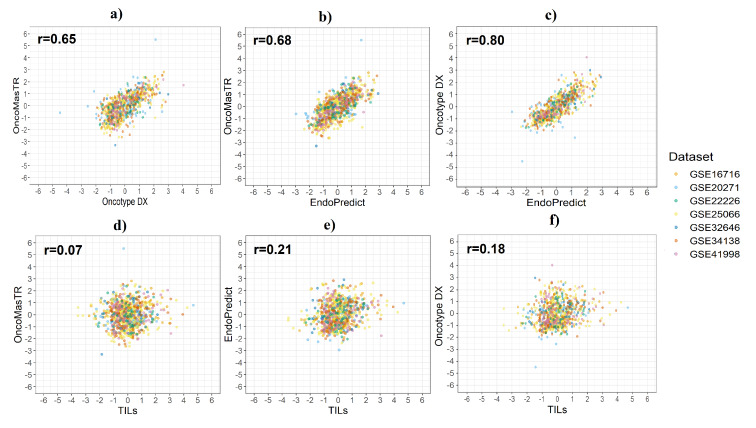
Correlation between risk scores by dataset. (**a**) OM vs. RS; (**b**) OM vs. EP; (**c**) RS vs. EP; (**d**) OM vs. TILs; (**e**) EP vs. TILs; (**f**) RS vs. TILs.

OM, RS and EP were moderately well correlated. However, there was very low correlation between TILs and the other three signatures. The GSE20271 dataset provided the lowest correlation in most cases. Figure 2 shows the results of the correlation analysis between risk score in the seven datasets combined.

### 3.2. Predicted Probability of pCR

Univariable, bivariable and trivariable logistic regression was used to estimate the relationship of OM, RS, EP and TILs with pCR. Table 3 contains a summary of the odds ratio (for a 1 standard deviation in risk score), 95% confidence intervals, and p-values for the relationship between risk scores and pCR.

Table 3 shows that the odds ratios of the signatures were similar in magnitude. OM, RS, EP and TILs were significant predictors of pCR, with TILs being less predictive. OM had an odds ratio of 1.66 with a 95% confidence interval of 1.29 to 2.16. EP had an odds ratio of 1.76 with a 95% confidence interval of 1.37 to 2.27. RS had an odds ratio of 1.84 with 95% confidence interval of 1.44 to 2.35. TILs had an odds ratio of 1.36 with a 95% confidence interval of 1.07 to 1.72.

**Figure 3:**
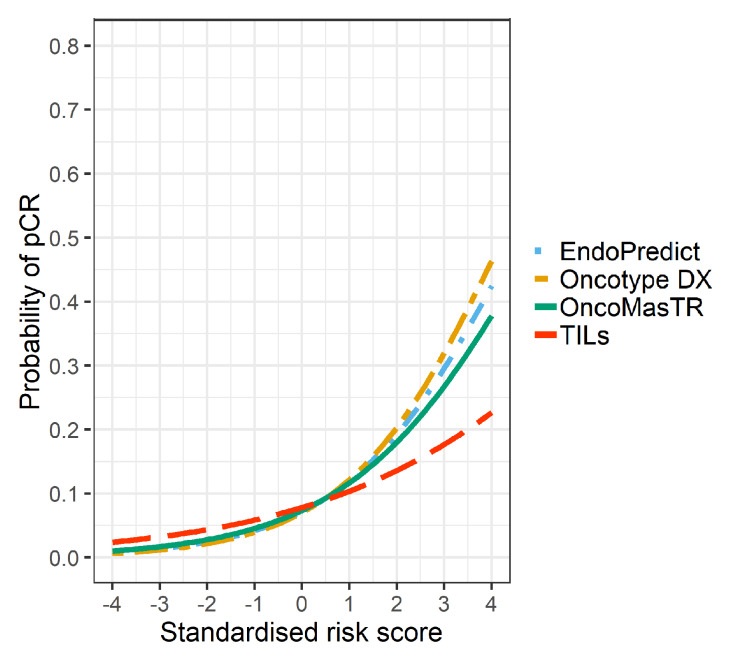
Predicted probability of pCR by risk score in 7 datasets (5 datasets missing ZNF367).

**Figure 4:**
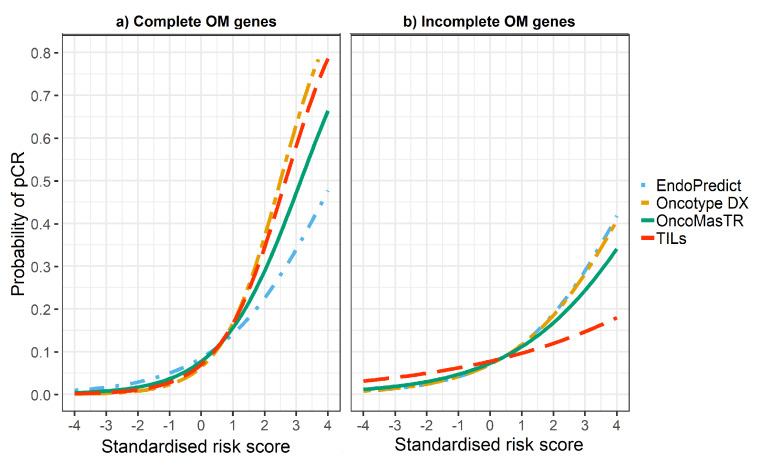
Predicted probability of pCR by risk score in (**a**) datasets with complete OM genes (2 datasets); (**b**) datasets with incomplete OM genes (5 datasets missing ZNF367).

**Figure 5:**
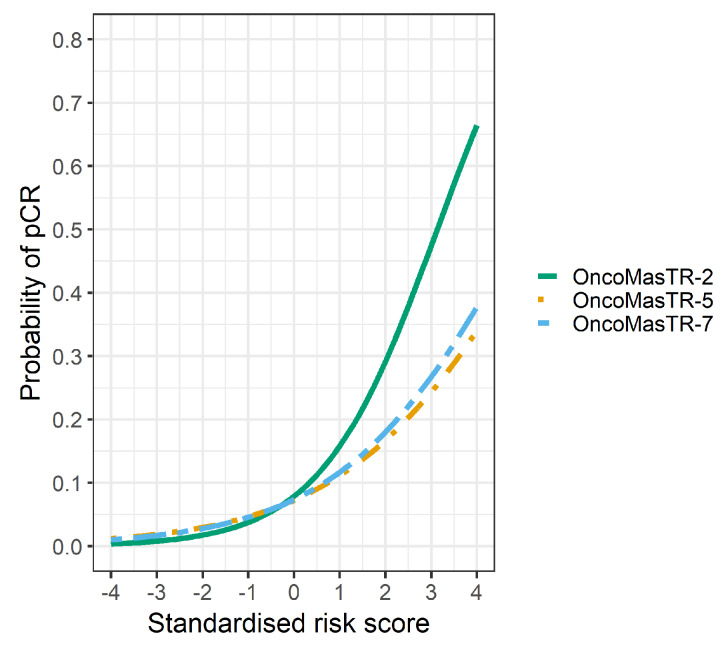
Predicted probability of pCR by OM risk score using different dataset groups. Green line complete OM genes (2 datasets); Orange line incomplete OM genes (5 datasets missing ZNF367); Blue line combined datasets (7 datasets).

The univariable odds ratios for OM, RS, EP and TILs were similar when controlled for dataset and/or other signatures (bivariable and trivariable models). Accordingly, we based our predicted probabilities on univariable odds ratio for clarity of presentation. Plots of the predicted probability (Figure 3, Figure 4 and Figure 5) are derived from the univariable odds ratios in Table 3 and show that the probabilities of pCR increase as the risk scores increase, indicating that the risk scores predict response to chemotherapy. Figure 4 shows that signatures with fewer genes are more likely to be impacted by missing genes. As hypothesised, the predictive performance of OM is better in datasets with complete OM genes (Figure 5).

Residual deviance was used to assess how much TILs added to the other three risk scores. Residual deviance is a measure of goodness of fit of a model, a lower deviance means a better fit model and a 0 deviance is a perfect model. TILs added significant predictive value if the residual deviance decreased by >3.84 when TILs was added to a model (*p*(*chi-sq* > 3.84, df = 1) < 0.05) (see Table 4).

TILs added significant predictive value to OM and vice versa and the significant predictive values are increased when the model is adjusted by dataset. TILs does not add significant predictive value to RS and EP (*p* = 0.0630 and 0.0886 respectively). However, RS and EP added significant predictive value to TILs. Controlling for the differences between the datasets further decrease the residual deviance of OM + TILs, RS + TILs and EP + TILs. The results show that the models adjusted for dataset are a better fit than unadjusted models. Finally, the best fit model was RS + TILs + Dataset with a residual deviance of 419.60.

## 4. Discussion

Multi-gene expression prognostic assays are commonly used to aid clinical decision-making in early-stage ER-positive, HER2-negative breast cancer as they provide complementary prognostic information to clinico-pathologic features [3]. There are many assays available, including RS, EP, TILs, MammaPrint [29], PAM50 [30], Breast Cancer Index (BCI) [31], along with the IHC4 test that is immunohistochemically based [32]. The amount of prognostic information provided in relation to risk of recurrence at an early (0–5 years) and late (beyond five years) time-point varies across these tests [33]. While the majority of data regarding these signatures focuses on prognostic value in the context of a background of adjuvant hormone therapy, there is comparatively little data available relating to their use in predicting benefit to neoadjuvant chemotherapy.

In this paper, we evaluated a new multi-gene prognostic signature, OM. We compared three other multi-gene expression prognostic signatures (RS, EP and TILs) with OM and evaluated the correlation between the four signatures (OM, RS, EP and TILs). Furthermore, we assessed if OM can predict pCR to neoadjuvant chemotherapy, and we compared the OM predictive performance with RS, EP and TILs. Finally, we evaluated if TILs added significant predictive value to OM, RS and EP.

Our findings suggest that OM, RS, and EP are moderately well correlated across the seven datasets assessed, with an average Pearson’s correlation coefficient *r*≥0.65 between each pair of signatures (OM vs RS, OM vs EP, and RS vs EP). TILs demonstrated a lower correlation for all datasets with an average *r* ≤0.21. Our results suggest that OM, RS, EP and TILs were significant predictors of pCR to neoadjuvant chemotherapy in ER+, HER2- breast cancer, with odds ratios ≥1.36.

TILs added significant predictive value to OM with a residual deviance decrease. OM, RS and EP added significant predictive value to TILs and controlling for the differences between the datasets further decreased the residual deviance. The best fit model was RS+TILs+Dataset demonstrating that including additional features provide a better fit to the data.

However, the predicted probability of pCR of the four signatures is affected by missing genes. It is important to highlight that OM is a significant pCR predictor even when only two of its prognostic genes are used (excluding missing genes). Our hypothesis is that if more datasets with complete genes were available then the performance of OM could be improved.

## 5. Conclusions and Future Work

OM has been clinically validated in the TransATAC cohort and a subset of the TAILORx cohort and has shown superior prognostic performance to RS [7,8,9]. Here, we show that the signature is predictive of treatment response (i.e., pCR response to neoadjuvant chemotherapy treatment). Some of the main differences between OM, RS, EP, and TILs are the number of genes in the assay (60–genes TILs, 21–genes RS, 12−genes EP, 6–genes OM, when normalization genes are included) and the resulting cost. Some limitations of this study are the approximation of risk scores in micro-array data, the small number of pCR events in the dataset, and the missing genes effect.

Biomarkers that predict patient response to neoadjuvant chemotherapy offer the opportunity for personalised care, improved therapy response rates, reduced adverse effects and decreased costs of unnecessary treatment. In the future, we will extend this work by following two lines of investigation: (i) using more datasets thereby increasing the number of subjects evaluated and reducing the number of missing genes; and (ii) exploring new techniques which combine images with genetic information.

## Figures and Tables

**Table 1:** GEO datasets included in the analysis *.

GEO Dataset	Platform	Patients (N)	pCR (N)	Missing Genes			
				EP	RS	TILs	OM
GSE16716	Affymetrix Human Genome U133A Array	140	7			PTRPC, KLRK1, EOMES, KIR3DL2, XCL2, CD8B	ZNF367
GSE20271	Affymetrix Human Genome U133A Array	89	6			PTRPC, KLRK1, EOMES, KIR3DL2, XCL2, CD8B	ZNF367
GSE25066	Affymetrix Human Genome U133A Array	278	30			PTRPC, KLRK1, EOMES, KIR3DL2, XCL2, CD8B	ZNF367
GSE32646	Affymetrix Human Genome U133A Plus 2.0 Array	55	5			PTRPC, KLRK1, KIR3DL2, XCL2	
GSE34138	Illumina Human WG 6 v3.0 expression bead chip	119	4		MYBL2	PTRPC, KLRK1, TPSB2, XCL2, NCR1, FOXP3	ZNF367
GSE41998	Affymetrix Human U133A 2.0 Array	93	10			PTRPC, KLRK1, EOMES, KIR3DL2, XCL2, CD8B	ZNF367
GSE22226 GPL1708	Agilent 012391 Whole Human Genome Oligo Microarray G4112A (Feature Number version)	39	4	CCNB1	MYBL2	PTRPC, EOMES, TPSB2, TPSB1, MS4A2, KIR3DL2, CD3E	
		813	66				

* OM corresponds to the OncoMasTR score, RS corresponds to the Oncotype DX Recurrence Score, EP corresponds to the EndoPredict score and TILs corresponds to the Tumor Infiltrating Leukocytes signature.

**Table 2:** Correlation coefficient *r* among the four signatures * **.

Signatures	Overall *r*	Lowest *r* (Dataset)	Highest *r* (Dataset)
OM vs. RS	0.65	0.34 (GSE20271)	0.79 (GSE41998)
OM vs. EP	0.68	0.44 (GSE20271)	0.82 (GSE41998)
RS vs. EP	0.80	0.55 (GSE20271)	0.90 (GSE32646)
OM vs. TILs	0.07	0.01 (GSE20271)	0.17 (GSE32646)
EP vs. TILs	0.21	0.09 (GSE41998)	0.33 (GSE20271)
RS vs. TILs	0.18	0.12 (GSE41998)	0.28 (GSE20271)

* Overall corresponds to the average of the seven datasets; Lowest and Highest correspond to the minimum and maximum, respectively. ** OM corresponds to the OncoMasTR score, RS corresponds to the Oncotype DX Recurrence Score, EP corresponds to the EndoPredict score and TILs corresponds to the Tumor Infiltrating Leukocytes signature.

**Table 3:** Odds ratio (95% confidence intervals) for pCR by risk score * **.

Signature	Odds Ratio (95% CI)	*p*-Value	Model
Univariable Analysis			
OM	1.66 (1.29–2.16)	0.0001	OM
RS	1.84 (1.44–2.35)	<0.0001	RS
EP	1.76 (1.37–2.27)	<0.0001	EP
TILs	1.36 (1.07–1.72)	0.0099	TILs
Bivariable Analysis (adjusted for dataset or TILs)			
OM	1.68 (1.30–2.18)	<0.0001	OM + Dataset
RS	1.85 (1.45–2.37)	<0.0001	RS + Dataset
EP	1.77 (1.37–2.30)	<0.0001	EP + Dataset
TILs	1.37 (1.08–1.73)	0.0095	TILs + Dataset
OM	1.63 (1.26–2.12)	0.0002	OM + TILs
TILs	1.32 (1.04–1.66)	0.0226	
RS	1.80 (1.40–2.31)	<0.0001	RS + TILs
TILs	1.26 (0.98–1.60)	0.0630	
EP	1.69 (1.31–2.20)	<0.0001	EP + TILs
TILs	1.23 (0.96–1.56)	0.0886	
Trivariable Analysis (adjusted for dataset and TILs)			
OM	1.65 (1.28–2.14)	0.0002	OM + TILs + Dataset
TILs	1.33 (1.04–1.69)	0.0199	
RS	1.81 (1.41–2.33)	<0.0001	RS + TILs + Dataset
TILs	1.27 (0.99−1.62)	0.0546	
EP	1.71 (1.32–2.23)	<0.0001	EP + TILs + Dataset
TILs	1.25 (0.97–1.59)	0.0748	

* Odds ratio is for a 1 standard deviation increase in risk score. ** OM corresponds to the OncoMasTR score, RS corresponds to the Oncotype DX Recurrence Score, EP corresponds to the EndoPredict score and TILs corresponds to the Tumor Infiltrating Leukocytes signature.

**Table 4:** Deviance statistic by model *.

Model	Null Deviance	df Null	LogLik	AIC	BIC	Deviance	df Residual
Univariable Analysis							
OM	457.95	812	−221.16	446.33	455.73	442.33	811
RS	457.95	812	−216.89	437.79	447.19	433.79	811
EP	457.95	812	−219.27	442.53	451.93	438.53	811
TILs	457.95	812	−225.80	455.59	464.99	451.59	811
Bivariable Analysis (adjusted for dataset or TILs)							
OM + Dataset	457.95	812	−215.84	447.68	485.29	431.68	805
RS + Dataset	457.95	812	−211.59	439.18	476.78	423.18	805
EP + Dataset	457.95	812	−213.94	443.89	481.49	427.89	805
TILs + Dataset	457.95	812	−220.55	457.09	494.70	441.09	805
OM + TILs	457.95	812	−218.65	443.30	457.41	437.30	810
RS + TILs	457.95	812	−215.22	436.44	450.55	430.44	810
EP + TILs	457.95	812	−217.86	441.71	455.82	435.71	810
Trivariable Analysis (adjusted for dataset and TILs)							
OM + TILs + Dataset	457.95	812	−213.21	444.43	486.73	426.43	804
RS + TILs + Dataset	457.95	812	−209.80	437.60	479.90	419.60	804
EP + TILs + Dataset	457.95	812	−212.40	442.80	485.11	424.80	804

* OM corresponds to the OncoMasTR score, RS corresponds to the Oncotype DX Recurrence Score, EP corresponds to the EndoPredict score and TILs corresponds to the Tumor Infiltrating. Leukocytes signature.

## Abbreviations

The following abbreviations are used in this manuscript:

BCI	Breast Cancer Index
CI	Confidence Interval
EP	EndoPredict
GEO	Gene Expression Omnibus
MTRs	Master Transcriptional Regulators
OM	OncoMasTR
pCR	Pathological Complete Response
RS	Oncotype DX
TILs	Tumor Infiltrating Lymphocytes
EI	Enterprise Ireland
IRC	Irish Research Council
SFI	Science Foundation Ireland
